# The effectiveness of artificial microbial community selection: a conceptual framework and a meta-analysis

**DOI:** 10.3389/fmicb.2023.1257935

**Published:** 2023-09-29

**Authors:** Shi-Rui Yu, Yuan-Ye Zhang, Quan-Guo Zhang

**Affiliations:** ^1^State Key Laboratory of Earth Surface Processes and Resource Ecology and MOE Key Laboratory for Biodiversity Science and Ecological Engineering, Beijing Normal University, Beijing, China; ^2^Key Laboratory of the Ministry of Education for Coastal and Wetland Ecosystems, College of the Environment and Ecology, Xiamen University, Xiamen, Fujian, China

**Keywords:** artificial selection, community trait, experimental evolution, microbial communities, selection strategy

## Abstract

The potential for artificial selection at the community level to improve ecosystem functions has received much attention in applied microbiology. However, we do not yet understand what conditions in general allow for successful artificial community selection. Here we propose six hypotheses about factors that determine the effectiveness of artificial microbial community selection, based on previous studies in this field and those on multilevel selection. In particular, we emphasize selection strategies that increase the variance among communities. We then report a meta-analysis of published artificial microbial community selection experiments. The reported responses to community selection were highly variable among experiments; and the overall effect size was not significantly different from zero. The effectiveness of artificial community selection was greater when there was no migration among communities, and when the number of replicated communities subjected to selection was larger. The meta-analysis also suggests that the success of artificial community selection may be contingent on multiple necessary conditions. We argue that artificial community selection can be a promising approach, and suggest some strategies for improving the performance of artificial community selection programs.

## Introduction

**Figure 1:**
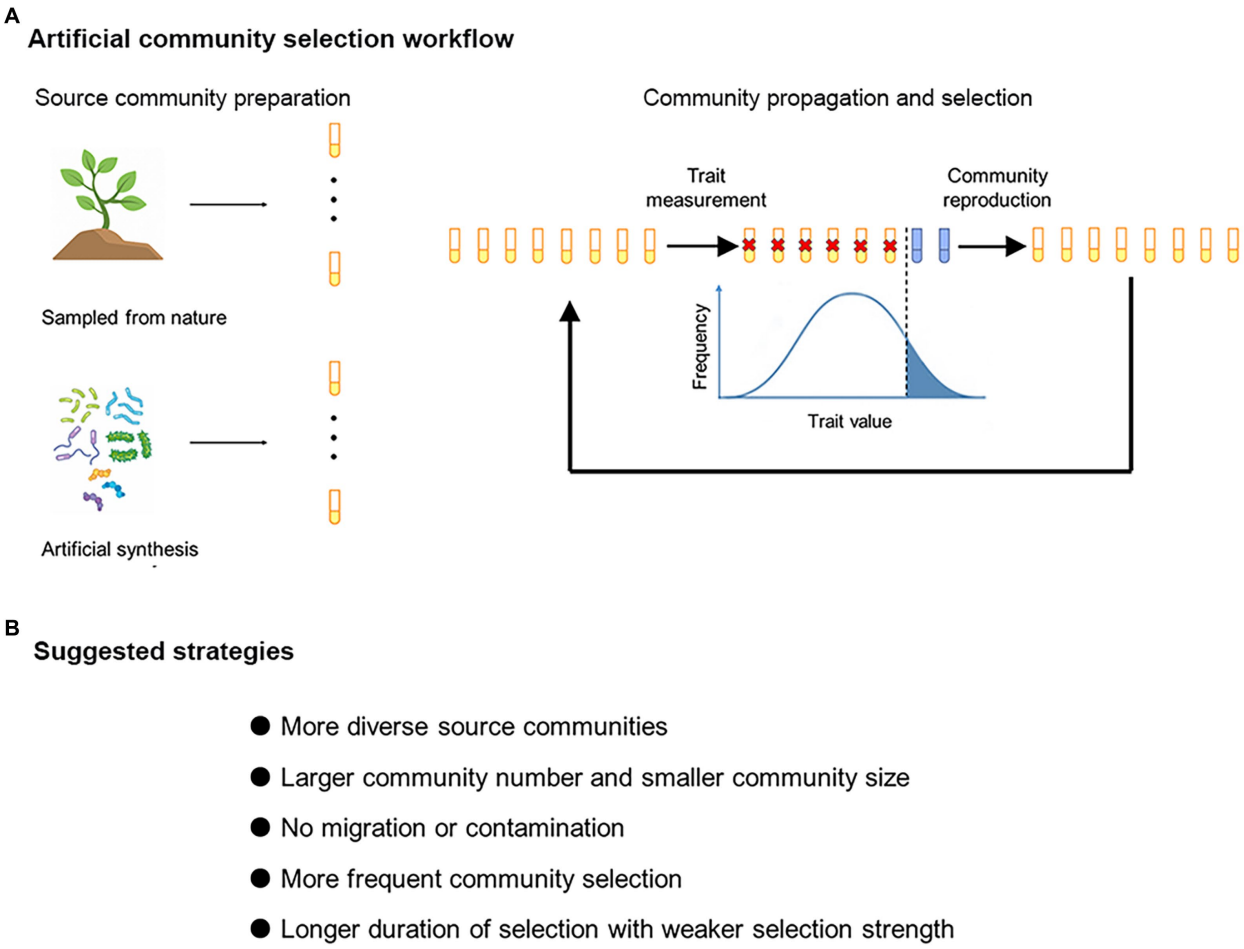
**(A)** Artificial community selection workflow. The source microbial communities can be obtained from natural environments or by artificial assembly. One selection line consists of multiple communities (here each test tube represents a community). The communities are propagated for multiple cycles, and those with extreme values of particular traits are chosen to “reproduce.” **(B)** Suggested selection strategies for improving the effectiveness of artificial community selection.

Artificial community selection is conceptually similar to crop breeding (Figure 1A). A selection line contains multiple ecological communities, which are propagated for a number of transfers. At each transfer, a proportion of communities within a selection line would be chosen to “reproduce,” i.e., to establish the next generation of communities. Selection of communities with extreme values of particular traits (e.g., greater respiration rate) to contribute to the next generation may result in heritable changes in community traits (Goodnight, 2000; Swenson et al., 2000b). In experimental studies, the artificial selection treatments are usually accompanied with random-selection or no-selection controls (Supplementary Figure S1). Successful artificial selection will manifest itself if a target community trait is changed to a greater magnitude under artificial selection than under control treatments (Swenson et al., 2000a,b). Typical examples of target community traits include higher capacity of degrading particular pollutants (Swenson et al., 2000a) and facilitation of host plant growth (Swenson et al., 2000b).

Artificial community selection has recently attracted much attention in applied microbiology. One possible reason is that many ecological functions can only be performed by multiple-species microbial communities. For example, decomposition of many complex organic materials may require combined actions of different enzymes secreted by different microbial taxa (Wolfaardt et al., 1994; Wagner-Döbler et al., 1998; Hubert et al., 1999). Also importantly, many microbes are difficult to isolate and to grow as pure cultures (Alain and Querellou, 2009; Overmann et al., 2017). Due to these challenges, implementing artificial selection on microbial communities could be a promising alternative to bottom-up assembling of communities. The success of artificial community selection cannot be taken for granted (Arora et al., 2020; Sánchez et al., 2021). A line of recent research has addressed what selection strategies may promote the efficiency of community selection; for example, it was suggested that migration (contamination) among communities should be avoided; and selection intensity should not be very high (Arias-Sánchez et al., 2019; Xie et al., 2019; Chang et al., 2021; Xie and Shou, 2021). However, a more comprehensively understanding of the determinants of the effectiveness of community selection is still lacking.

Successful high-level selection usually requires overcoming the impacts of individual-level natural selection, as suggested by the “tragedy of the commons” (Rankin et al., 2007; MacLean, 2008; Anten and Vermeulen, 2016). Multilevel selection studies suggested a number of conditions that may increase the power of high-level selection. For example (within-species) group selection is expected to be more effective when heritable variance among groups, relative to within groups, is greater (Leigh Jr, 2000; Wade, 2016). This could be interpreted as the latter fueling individual-level natural selection that may oppose group-level selection. It was also suggested that faster frequency of group selection increases its effectiveness, as individual-level natural selection would override group selection if group “longevity” is much larger compared with individuals (Levins, 1970). Multilevel selection theory suggests high-level selection may be powerful (Griffing, 1981; Goodnight, 1990a,b; Goodnight and Stevens, 1997). Experimental studies, particular crop breeding programs, demonstrated the potential for high-level selection to effectively change organism phenotypes (Wade, 1977; McCauley and Wade, 1980; Wade, 1980, 1982; Goodnight, 1985; Goodnight, 1990a,b; Wade and Goodnight, 1991; Craig and Muir, 1996).

Here we formulize a series of hypotheses regarding the effectiveness of artificial microbial community selection, as a summary and extension of previous studies in this field. We then conduct a meta-analysis of published experiments, to examine whether artificial microbial community selection programs are generally promising and test several specific hypotheses regarding how particular selection strategies may influence the effectiveness of artificial community selection.

## The hypotheses

*Hypothesis 1*. Artificial selection of microbial communities is more effective when the target phenotype is a trait expressed by microbes rather than their host animals or plants. For example, selection on the respiration rate of microbial communities can be more effective than that on the leaf greenness of their host plants. This is because, in the former case, heritable variation of the trait under selection originates entirely from the microbial communities, whereas in the latter case, such variation may be only partially attributable to the microbes.

*Hypothesis 2*. Artificial community selection is more effective when the variance among communities, relative to that within communities, is greater. This is a simple extension of knowledge of group selection (Leigh Jr, 2000; Wade, 2016). This leads to a suggestion that including more diverse source communities in artificial community selection programs could improve its effectiveness.

*Hypothesis 3*. Artificial community selection is more effective when the number of communities per selection line relative to the number of individuals in a community is larger. This could increase the variance among communities relative to within communities, and thus increase heritable variation in the trait under selection (Traulsen and Nowak, 2006). Moreover, the effect of drift would be less important relative to selection when the number of communities within selection lines is larger. The number of communities is amenable to manipulation in experimental studies, but the number of individuals within a community may not. Thus, a testable prediction of this hypothesis is that the effectiveness of community selection is overall greater in experiments with a larger number of communities per selection line.

*Hypothesis 4*. Migration among communities reduces the effectiveness of selection by decreasing variance among communities, similar to that gene flow reduces the effectiveness of group selection by reducing the variance among groups (Slatkin and Wade, 1978; Wade, 1982; Leigh Jr, 1983). Migration among communities takes place in the “migrant pool” approach in artificial microbial community selection programs, where communities chosen as “parent” communities are mixed before seeding the next generation of communities (Swenson et al., 2000b; Raynaud et al., 2019). This hypothesis predicts that this “migrant pool” strategy would have poorer performance than the “propagule” approach where no migration among communities is allowed.

*Hypothesis 5*. The effectiveness of selection is positively correlated with the intensity of selection for short-term selection, and they are expected to exhibit a negative correlation in long-term selection. This is because short-term selection acts on the existing genetic variation among communities within selection lines, while long-term selection also relies on newly generated genetic variation. Under weaker selection, a larger number of communities can contribute to the subsequent generations, which in turn accumulate more new mutations available for selection. The intensity of artificial selection is typically manipulated by the proportion of communities selected, and smaller selected proportion corresponds to higher intensity of selection. We suggest that weaker selection combined with longer-term artificial selection (more rounds of community propagation and selection) would cause more substantial changes in community traits.

*Hypothesis 6*. Community selection is more efficient at faster frequencies of community selection and propagation, relative to the recruitment of individual organisms within communities. Artificial community selection takes place only when it was carried out artificially, and organisms may reproduce more than one generation during a single community selection cycle; and we need to notice that lower-level natural selection takes place all the time as long as individual organisms have differential rates of reproduction and survival. Thus lower-level natural selection would limit the success of community selection to a larger extent when community “longevity” is greater. This logic is the same as that the power of group selection is increased by more frequent group-level selection (Levins, 1970).

## The meta-analysis

### Meta-analysis methods

We compiled experimental studies for the meta-analysis in April 2023 by searching in the Web of Science for research articles published from year 1936 to 2023, using the following keywords: (bacteria* OR microbi*) AND (communit* OR ecosystem*) AND (“artificial selection”) NOT (natur* OR male* OR female* OR m?n OR wom?n OR intelligence), while refining for the direction of Microbiology or Environment Sciences Ecology research. There were 52 articles associated with these keywords, from which we screened for experiments suitable for our analysis. We also included several publications not captured by the search, particularly those referred to by Chang et al. (2021) and Sánchez et al. (2021). The following criteria were used to choose individual articles for the meta-analysis. First, the study system should be multiple-species microbial communities (species richness >3), rather than single-species cultures or highly simple communities. Second, there must be both artificial selection treatments and appropriate control (random or no-selection lines) treatments of comparable sizes of the selection lines, for which data of community-level phenotype values at the end of study were available. Third, the communities must have been propagated for over 3 transfers. One individual article could include more than one independent selection experiments (one particular artificial selection regime and a control regime would constitute one experiment). A total of 13 experiments from seven publications were finally included in our analysis (Supplementary Figure S2; Table 1, and see more details in Supplementary Tables S1, S2). The control treatments in all the 13 experiments were random-selection lines.

**Table 1:** Summary of artificial community selection experiments included in the meta-analysis.

Experiment	Target phenotype	Migration	No. communities per selection line	Selected proportion	No. selection lines	Effect size (lnRR)	Variance in effect size
Swenson et al. (2000a)	Degradation of 3-chloroaniline	Yes	15	0.2	4	0.001784	0.002274
Blouin et al. (2015)	CO_2_ emissions	Yes	30	0.1	6	0.317321	0.000303
Wright et al. (2019): experiment 1, 9 days	Chitinase activity	Yes	30	0.1	1	0.040445	0
Raynaud et al. (2019): migrant pool, high line	Microbial biomass	Yes	30	0.1	1	−0.04832	0
Raynaud et al. (2019): propagule, high line	Microbial biomass	No	10	0.1	3	−0.01706	0.000303
Raynaud et al. (2019): migrant pool, low line	Microbial biomass	Yes	30	0.1	1	0.081569	0
Raynaud et al. (2019): propagule, low line	Microbial biomass	No	10	0.1	3	0.140848	0.000303
Arora et al. (2020): high sugar	Host insect eclosion time	No	3	0.33	10	−0.00301	0.0001
Arora et al. (2020): low sugar	Host insect eclosion time	No	3	0.33	10	0.01507	4.67E-05
Chang et al. (2020): amylolytic activity	Amylolytic activity	No	24	0.16	1	0.494901	0
Chang et al. (2020): cross-feeder	Degradation of cycloheximide	No	92	0.25	1	2.202364	0
Jacquiod et al. (2022): high line	Host plant leaf greenness	Yes	20	0.15	3	0.019776	0.000303
Jacquiod et al. (2022): low line	Host plant leaf greenness	Yes	20	0.15	3	−0.0114	0.000303

We obtained the mean and standard deviation values of community traits for the control and artificial selection lines at the end of each experiment. Community traits studied in those experiments included microbial biomass, microbial respiration rate, activity of certain enzymes, capacity of degrading certain materials (which may be measured as how the end products support the growth of particular reference microbial strains), or growth performance of host animals or plants (e.g., eclosion time of host insets, or leaf greenness of host plants) (Table 1). For articles of which raw data were not available, we extracted data based on graphs, using Getdata graph Digitizer version 2.2.6. We calculated the effect size as a natural logarithm transformed response ratio, lnRR = $\ln\;\left(\bar{X_{a}}/\bar{X_{c}}\right)$ for each experiment that selected for increased phenotype values (“high lines”), and $-\ln\;\left(\bar{X_{a}}/\bar{X_{c}}\right)$ for that selected for decreased phenotype values (“low lines”), where $\bar{X_{a}}$ and $\bar{X_{c}}$ were mean values of community phenotype (e.g., total biomass) of the artificial selection lines and controls at the end of the selection experiment, respectively. The phenotype data were all original measurements, but not transformed data (imagine an experiment reporting pH values, the calculation should be based on H^+^ concentration, but not pH). Variance of effect size was calculated as Var = $\left[\left({{SD}_{c}}^{2}\right)/N_{c}\;{\left(\bar{X_{c}}\right)}^{2}\right]+\left[{\left({SD}_{a}\right)}^{2}/N_{a}\;{\left(\bar{X_{a}}\right)}^{2}\right]$ , where ${SD}_{c}$ was the standard deviation of the community phenotype values among the control lines, ${SD}_{a}$ for the artificial selection lines, and *N*_c_ and *N*_a_ represented the number of selection lines under the control and artificial selection treatments, respectively. When standard errors (SE) were reported, standard deviation was calculated as SD = $SE*\mathrm{sqrt}\;\left(X\right)$ . For studies where the SD or SE was not given, the missing variance was substituted with the median of the given lnRR variances (Corrêa et al., 2012).

Although a number of factors may influence selection effect size, information about some of them cannot be obtained or evaluated (e.g., the variance among source communities and the number of individual organisms within communities). We were able to obtain information about the following attributes from the 13 experiments, and study their relation with the effect size of artificial selection: microbial vs. host phenotype, presence of migration among communities (migrant pool vs. propagule approach), size of selection lines (average number of communities per selection line), and selected proportion (i.e., percentage of communities chosen to “reproduce” at each transfer) (Table 1). Those four factors were defined as moderators in the following analysis. We used the “metafor” package 3.8-1 from R version 4.2.3 for statistical analysis (Viechtbauer, 2010; R Core Team, 2023). The main effects of the four moderators were examined using a “*rma*” model with maximum likelihood method; and we performed deletion test for the significance of each main effect, comparing the 4-variable model with models excluding single moderators. Interaction effects were not considered due to the relatively small sample size (*n* = 13).

The robustness of the detected moderator effects was examined by sensitivity analyses of any disproportionate effects of single, independent experiments. Sensitivity analyses were carried out for every moderator variable that showed a significant effect on the effect sizes (“*leave-one-out*” function in the “metafor” package) (Viechtbauer, 2010; R Core Team, 2023). We extracted the means and confidence intervals (CIs) of the effect sizes and compared them to the model for the non-excluded studies. Those experiments whose CIs did not include the mean of the non-excluded model were identified as having a disproportionate effect and needed to be excluded.

### Meta-analysis results

**Figure 2:**
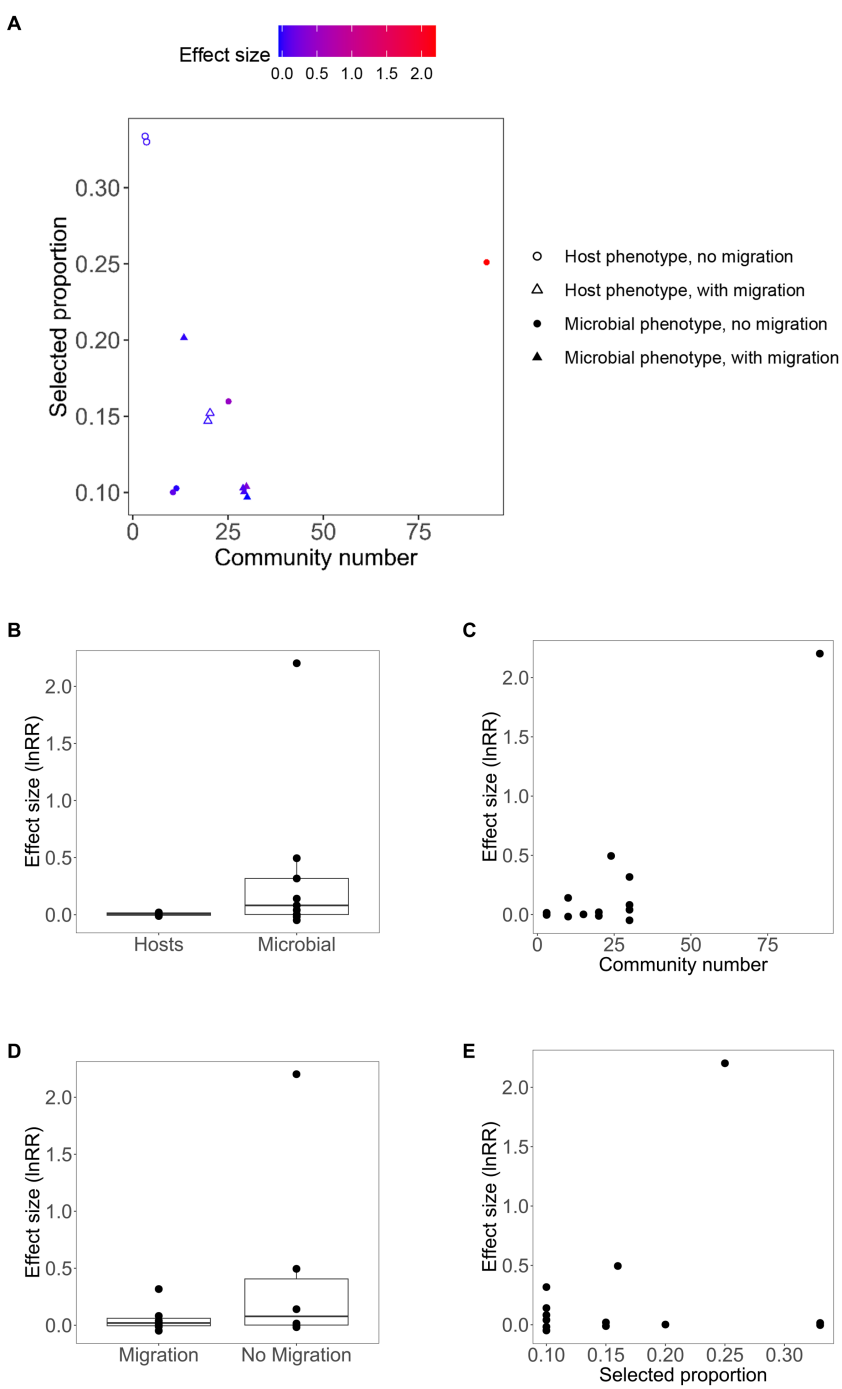
**(A)** Relationship between effect size of artificial community selection experiments and four moderators. Effect size values are mapped to point color on the selected proportion-community size plot, with point shape defined by two categorial moderators (microbial vs. host phenotype, and presence of migration among communities). **(B–E)** Relationships between effect sizes and single moderators.

A total of 13 effect sizes of artificial microbial community selection were included in this meta-analysis. Those effective size values ranged from −0.0483 to 2.2023. Eight out of the 13 effect sizes were positive (Table 1). The mean effect size was 0.2488 ± 0.3172 (95% confidence interval), which was not significantly different from zero (*p* = 0.1241, *n* = 13). We were able to test for the importance of four factors for the effect size of artificially selection experiments: microbial vs. host phenotype, migration, community number and selected proportion. Figure 2 shows the relationship between effect sizes and the four moderators. Among those four moderators, community number and the presence of migration were statistically significant predictors (Table 2). In the 4-variable model, specifically, effective size was smaller in experiments with migration among communities, relative to no migration (*p* < 0.0001; estimated difference in intercepts: −0.3828 ± 0.1397); and was greater in experiments with larger community numbers per selection line (*p* < 0.0001; estimated slope: 0.0246 ± 0.0029). Effect size did not significantly differ between experiments that targeted microbial phenotypes vs. host phenotypes (*p* = 0.9847; estimated difference in intercepts: −0.0018 ± 0.1819), and did not significantly change with selected proportion (*p* = 0.1488; estimated slope: 0.7950 ± 1.0792).

**Table 2:** Significance test for moderator effects analyzed in the meta-analysis, based comparisons between a model including the main effects of all the four moderators and those with single moderators excluded.

	df	ΔAIC	ΔBIC	Chi-squared	*p*
Microbial vs. host phenotype	(1, 5)	1.9997	2.5646	0.0004	0.985
Community number	(1, 5)	−38.3207	−37.7558	40.3208	**<0.001**
Migration	(1, 5)	−13.1709	−12.6059	15.1709	**<0.001**
Selected proportion	(1, 5)	0.0497	0.6147	1.9503	0.163

ΔAIC, ΔBIC, and Chi-squared represent the changes in the Akaike information criterion and Bayesian information criterion, and the statistics of the likelihood ratio tests.

Significant *p*-values (*p* < 0.05) are in bold.

The sensitivity analysis did not suggest excluding any effect size from analysis (Supplementary Table S3). However, we note that an extremely large effect size, 2.2024 (Chang et al., 2020: cross-feeder) differed substantially from the others that ranged from −0.0483 to 0.4949. When this particular experiment was excluded from analysis, the overall effect size was 0.0862 ± 0.0879, marginally non-significantly different from zero (*p* = 0.0546, *n* = 12); and the results from analysis regarding the moderator effects were not qualitatively different from the above analysis (Supplementary Table S4).

## Discussion

Improvement of ecological functions driven by microbes often require community-level manipulation (Swenson et al., 2000b; Day et al., 2011; Mueller and Sachs, 2015; Doulcier et al., 2020). However, artificial selection at the community level may not always lead to desirable responses. Theoretical and experimental work is needed to figure out what conditions may generally increase the efficiency of artificial community selection (Sánchez et al., 2021). The hypotheses formulized in the present study are within this line of effort (Figure 1B).

Well-designed artificial microbial community selection experiments are still scarce; in particular, a number of studies lacked appropriate control treatment, i.e., random-selection or no-selection lines that have comparable community numbers as artificial selection lines. Results of our meta-analysis should certainly be interpretated with caution, particularly because of the small sample size. These effect sizes were highly variable; and the mean value was not significantly different from zero, though 8 out of 13 experiments showed positive effective sizes. It is certainly possible that the small sample size (*n* = 13) has limited statistical power to detect significant overall effect here.

While not able to test all the hypotheses outlined above in this article, our meta-analysis addresses the importance of four factors supposedly relevant to the success of artificial community selection. The effectiveness of artificial selection was greater when there is no migration among communities within selection lines, and the community number per selection line is larger. Both of those factors may increase the variance among vs. within communities (the operational unit of artificial selection). There was no significant difference between experiments that targeted microbial phenotypes and those targeted host phenotypes; and selected proportion was not a significant predictor for effect size of artificial selection experiments. We were unable to extract quantitative information about phenotypic variance among communities, or that within communities, from the published experiments, nor information about selection frequency on the scale of generation numbers of organisms (selection frequency measured on the basis of absolute time units was available). Thus, our Hypothesis 2 and 6 cannot be tested here. Experimental studies, but not meta-analyses, might be more appropriate for testing those two hypotheses, as variance among communities and selection frequency may be manipulated in a particular experiment, but may not be comparable across different study systems.

There was one outstandingly large effect size, 2.2023 (with the remaining ones ranging from −0.0483 to 0.4949); this effect size value corresponds to artificial selection being 8.05-fold (e^2.2023^–1) more effective than random selection in altering community phenotypes. This experiment selected for greater capacity of degrading the antifungal cycloheximide (Chang et al., 2020: cross-feeder experiment). The design of this experiment was consistent with several hypothesized conditions for successful artificial community selection. In particular, it was distinct from the other experiments by having a combination of large size of selection lines (community number per selection line) and large selected proportion (Figure 2A). Moreover, the target trait was one expressed by microbes but not hosts. Community size was expected to be relatively small (microbes grown in 500 μL of 0.2% citrate-M9 media with 200 μg/mL cycloheximide which was resource-poor and unlikely to support large population sizes). There was not migration among communities. It may also be true that the initial variance among communities was large: 12 environmental samples as source communities. This suggests a possibility that an “Anna Karenina” principle may apply to artificial community selection: “success actually requires avoiding many separate possible causes of failure” (Diamond, 1999). Thus, multiple factors that may determine the effectiveness of artificial community selection should be studied in a more comprehensive manner in future. An alternative, non-mutually exclusive, explanation for the success of this particular experiment is that responses to selection are usually large when organisms are faced with novel environments; this is because genetic variation with great fitness effects is available when organisms are poorly adapted to the environment. And adaptation usually decelerates over time due to diminishing-returns epistasis (de Visser et al., 1999; Bell, 2007; Kassen, 2014). Therefore, experiments that select for faster degradation of novel pollutants may show large effect size. Note that this cannot be always true; an experiment selecting for degradation of 3-chloroaniline observed very weak response to selection (Table 1; Swenson et al., 2000a).

In conclusion, we suggest that artificial community selection could be a promising approach to improving ecological functions, although the success of artificial community selection may be contingent on certain conditions, or a combination of multiple conditions. It pays off to figure out the conditions that enhance the effectiveness of community-level selection; and future studies that carefully examine knowledge of multilevel selection may provide valuable insights into the recipe of successful artificial community selection.

## Data availability statement

The original contributions presented in the study are included in the article/Supplementary material, further inquiries can be directed to the corresponding author.

## Author contributions

S-RY: Formal analysis, Investigation, Writing—original draft. Y-YZ: Formal analysis, Writing—original draft. Q-GZ: Conceptualization, Formal analysis, Funding acquisition, Investigation, Project administration, Supervision, Writing—original draft.

## Funding

The author(s) declare financial support was received for the research, authorship, and/or publication of this article. This work was supported by the National Natural Science Foundation of China (32371687, 31725006), the 111 project (B13008), and the Fundamental Research Funds for the Central Universities of China.

## Conflict of interest

The authors declare that the research was conducted in the absence of any commercial or financial relationships that could be construed as a potential conflict of interest.

## Publisher’s note

All claims expressed in this article are solely those of the authors and do not necessarily represent those of their affiliated organizations, or those of the publisher, the editors and the reviewers. Any product that may be evaluated in this article, or claim that may be made by its manufacturer, is not guaranteed or endorsed by the publisher.

## Supplementary material

SUPPLEMENTARY TABLE S1 Summary of published articles captured by our literature search.

SUPPLEMENTARY TABLE S2 Detailed information about the studies used in our meta-analysis.

SUPPLEMENTARY TABLE S3 Results of sensitivity analysis for each study included in the meta-analysis.

SUPPLEMENTARY TABLE S4 Significance test for moderators analyzed in this meta-analysis with an outstandingly large effect size excluded (Chang et al., 2020): cross-feeder).

SUPPLEMENTARY FIGURE S1 A graphical illustration of the artificial selection **(A)** random-selection control **(B)** and no-selection control **(C)** The random-selection protocol chooses a proportion of communities randomly (regardless of their traits) to contribute to the next generation of communities. Under the no-selection control, every community would contribute to one offspring community at each round of community propagation.

SUPPLEMENTARY FIGURE S2 Preferred Reporting Items for Systematic reviews and Meta-analysis (PRISMA) diagram that shows an overview of the study selection process for our meta-analysis.

SUPPLEMENTARY FIGURE S3 Relationship between effect sizes of experiments and the four moderators after an outstandingly large effect size (Chang et al., 2020): cross-feeder) was excluded.
