# Supplementary material for: The effectiveness of artificial microbial community selection: a conceptual framework and a meta-analysis
Source: Front Microbiol. 2023 Sep 29;14:1257935. doi: 10.3389/fmicb.2023.1257935 (PMC10570731; doi:10.3389/fmicb.2023.1257935)
Supplement: SUPPLEMENTARY TABLE S4 — Significance test for moderators analyzed in this meta-analysis with an outstandingly large effect size excluded (Chang et al., 2020): cross-feeder). [file Table_4.docx]

**Supplementary Table 4.** Significance test for moderators analyzed in this study with an outstandingly large effect size excluded (Chang et al. (2020): cross-feeder). The analysis was based comparisons between a model including the main effects of the four moderators and those with single moderators excluded. ΔAIC, ΔBIC and Chi-squared represent the changes in the Akaike information criterion and Bayesian information criterion, and the statistics of the likelihood ratio tests. Significant *P*-values (*P* < 0.05) are in bold. The four-moderator model suggested that effect size increased with increasing community number (estimated slope: 0.0194 ± 0.0061); and selection sizes were smaller in experiment with migration among communities, relative to no migration (estimated difference in intercepts: -0.3205± 0.1006).

|  | df | ΔAIC | ΔBIC | Chi-squared | *P* |
| --- | --- | --- | --- | --- | --- |
| Microbial versus host phenotype | (1, 5) | 1.9703 | 2.4552 | 0.0297 | 0.863 |
| Community number | (1, 5) | -5.252 | -4.7671 | 7.252 | **0.007** |
| Migration | (1, 5) | -53504 | -4.8656 | 7.3505 | **0.007** |
| Selected proportion | (1, 5) | 1.2333 | 1.7181 | 0.7668 | 0.381 |
