# Supplementary figures and images for: The effectiveness of artificial microbial community selection: a conceptual framework and a meta-analysis
Source: Front Microbiol. 2023 Sep 29;14:1257935. doi: 10.3389/fmicb.2023.1257935 (PMC10570731; doi:10.3389/fmicb.2023.1257935)

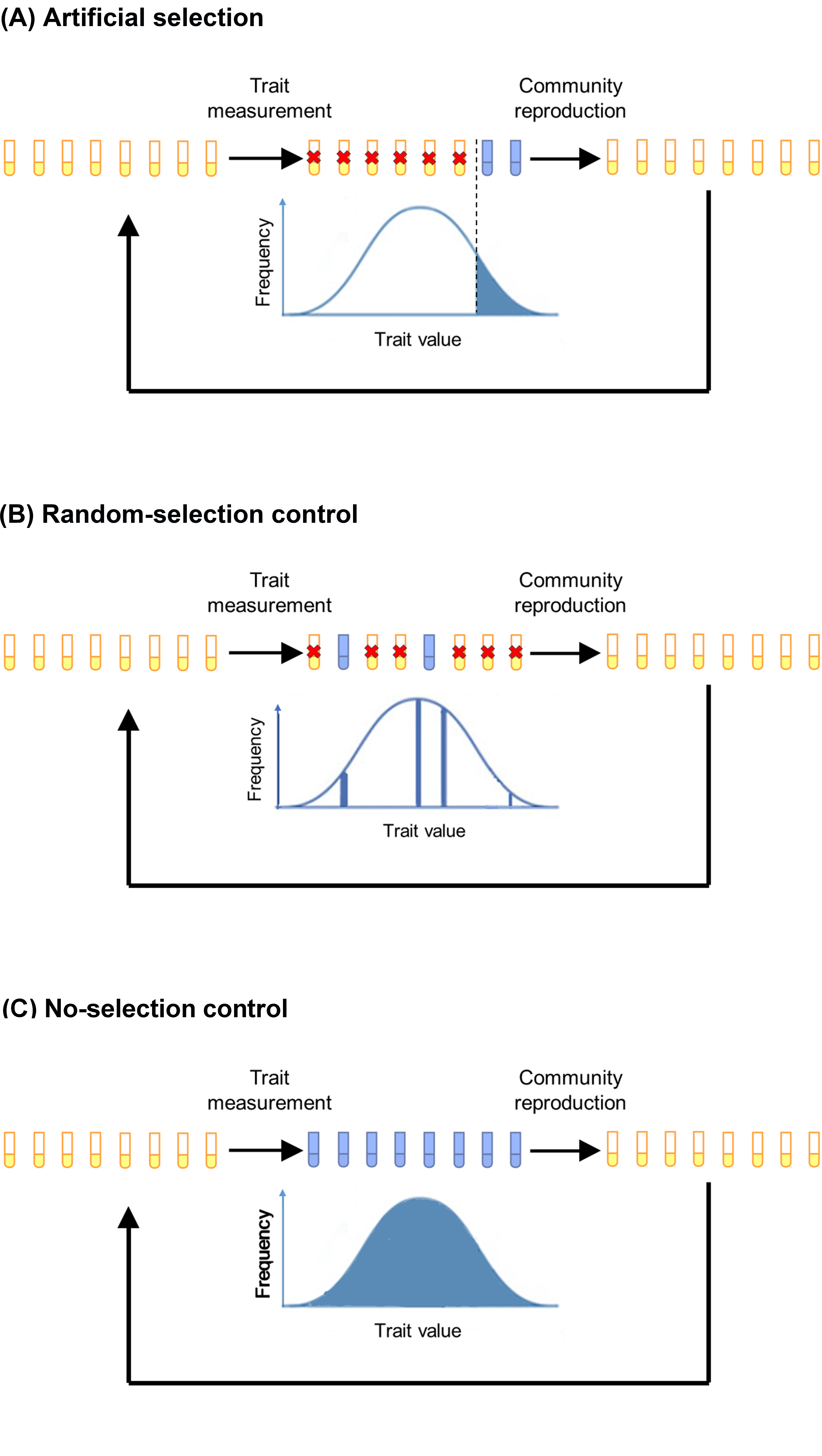

Supplement: SUPPLEMENTARY FIGURE S1 — A graphical illustration of the artificial selection (A) random-selection control (B) and no-selection control (C) The random-selection protocol chooses a proportion of communities randomly (regardless of their traits) to contribute to the next generation of communities. Under the no-selection control, every community would contribute to one offspring community at each round of community propagation. [file Image_1.tif]

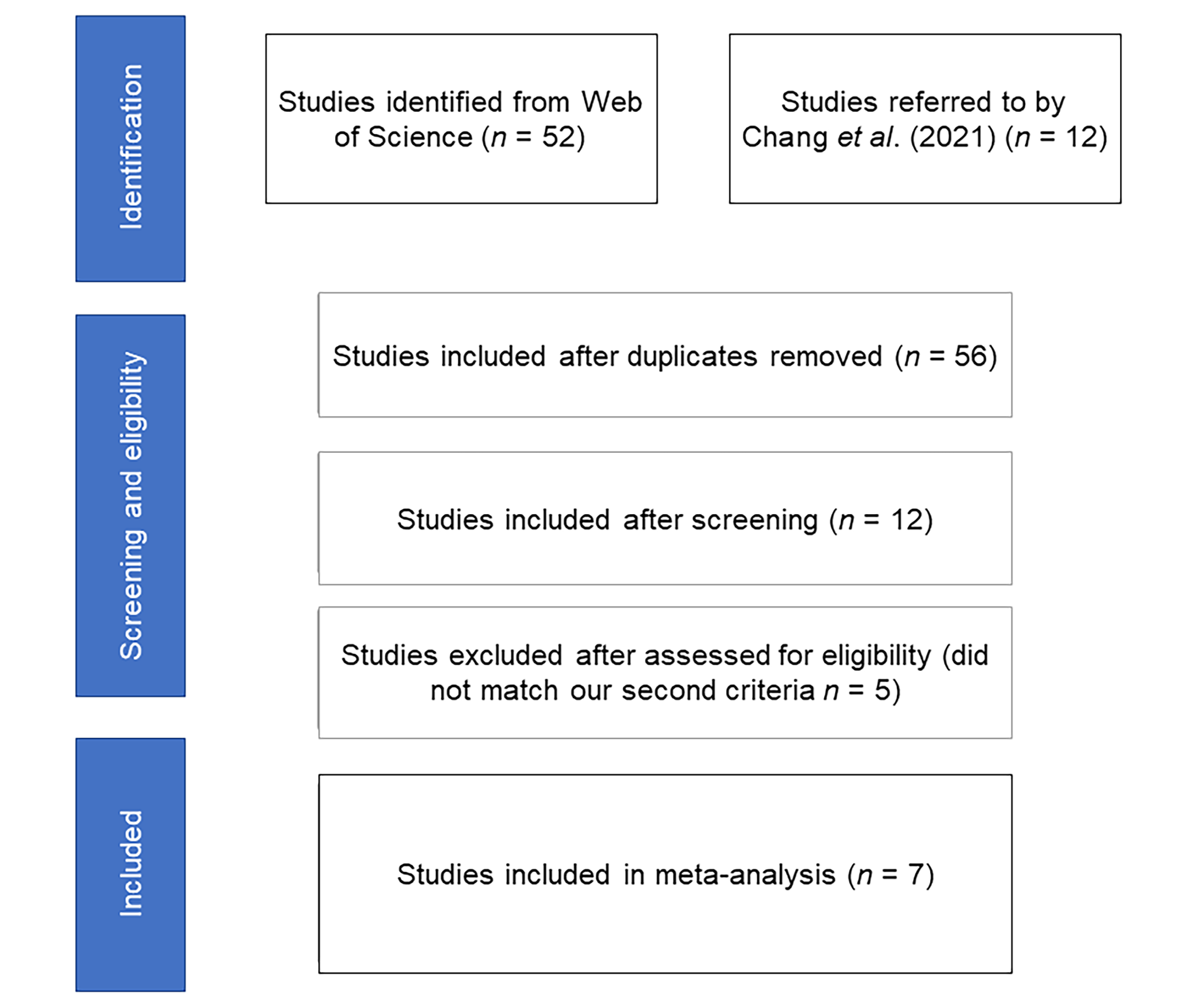

Supplement: SUPPLEMENTARY FIGURE S2 — Preferred Reporting Items for Systematic reviews and Meta-analysis (PRISMA) diagram that shows an overview of the study selection process for our meta-analysis. [file Image_2.tif]

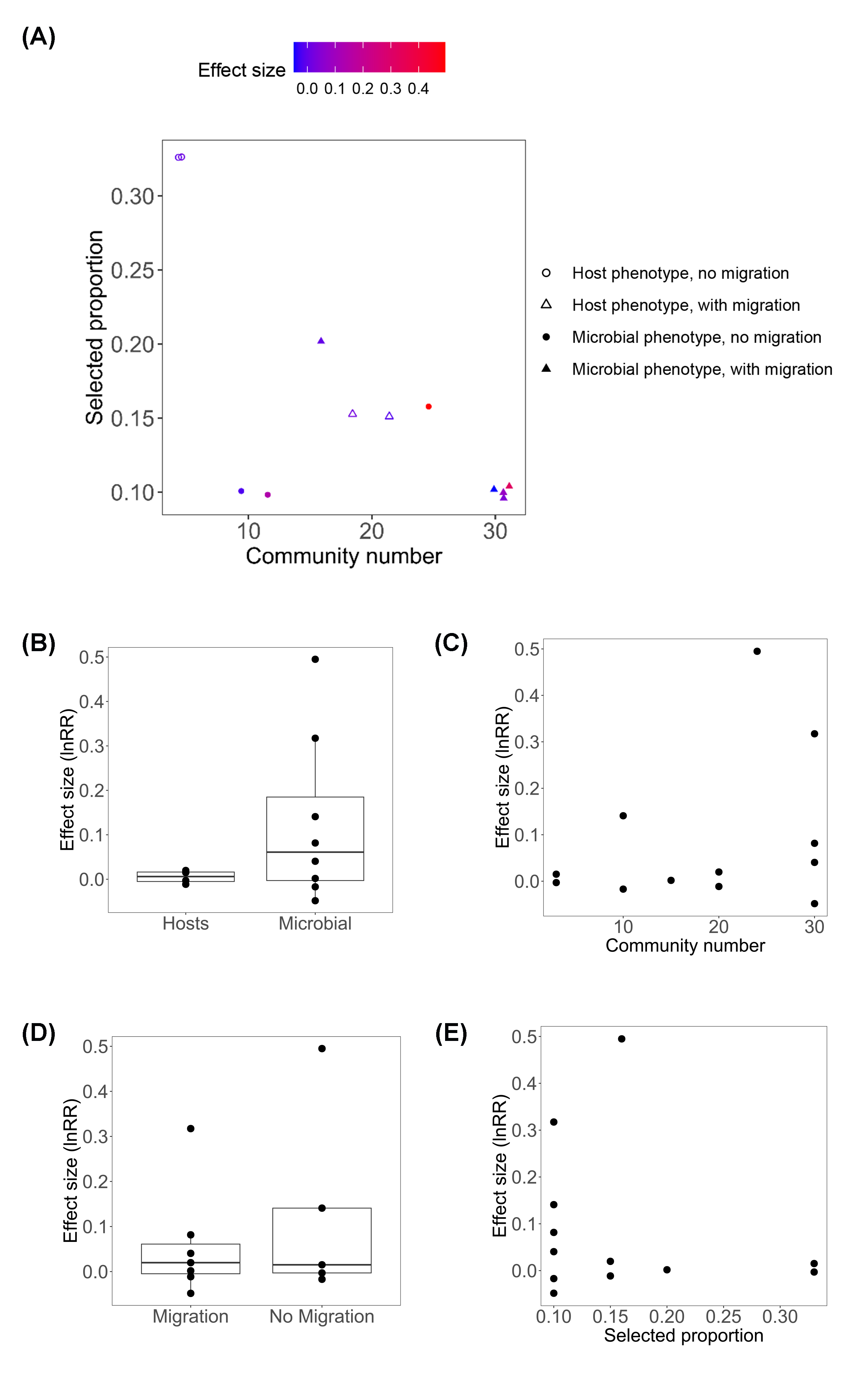

Supplement: SUPPLEMENTARY FIGURE S3 — Relationship between effect sizes of experiments and the four moderators after an outstandingly large effect size (Chang et al., 2020): cross-feeder) was excluded. [file Image_3.tif]
