# Supplementary material for: The effectiveness of artificial microbial community selection: a conceptual framework and a meta-analysis
Source: Front Microbiol. 2023 Sep 29;14:1257935. doi: 10.3389/fmicb.2023.1257935 (PMC10570731; doi:10.3389/fmicb.2023.1257935)
Supplement: Supplementary file 8 [file Table_1.DOCX]

Supplementary information

**Artificial community selection in microbes: a conceptual framework and a meta-analysis**

**Author Information**

**Shi-Rui Yu^1^, Yuan-Ye Zhang^2^, Quan-Guo Zhang^1^***

^1^State Key Laboratory of Earth Surface Processes and Resource Ecology and MOE Key Laboratory for Biodiversity Science and Ecological Engineering, Beijing Normal University, Beijing 100875, China

^2^Key Laboratory of the Ministry of Education for Coastal and Wetland Ecosystems, College of the Environment and Ecology, Xiamen University, Xiamen, Fujian 361102, China

*** Correspondence:**Quan-Guo Zhang
[zhangqg@bnu.edu.cn](mailto:zhangqg@bnu.edu.cn)

**Table S1** Significance test for moderators analyzed in this study with an outstandingly large effect size excluded (Chang et al. (2020): cross-feeder). The analysis was based comparisons between a model including the main effects of the four moderators and those with single moderators excluded. ΔAIC, ΔBIC and Chi-squared represent the changes in the Akaike information criterion and Bayesian information criterion, and the statistics of the likelihood ratio tests. Significant *P*-values (*P* < 0.05) are in bold. The four-moderator model suggested that effect size increased with increasing community number (estimated slope: 0.0194 ± 0.0061); and selection sizes were smaller in experiment with migration among communities, relative to no migration (estimated difference in intercepts: -0.3205± 0.1006).

|  | df | ΔAIC | ΔBIC | Chi-squared | *P* |
| --- | --- | --- | --- | --- | --- |
| Microbial versus host phenotype | (1, 5) | 1.9703 | 2.4552 | 0.0297 | 0.863 |
| Community number | (1, 5) | -5.252 | -4.7671 | 7.252 | **0.007** |
| Migration | (1, 5) | -53504 | -4.8656 | 7.3505 | **0.007** |
| Selected proportion | (1, 5) | 1.2333 | 1.7181 | 0.7668 | 0.381 |


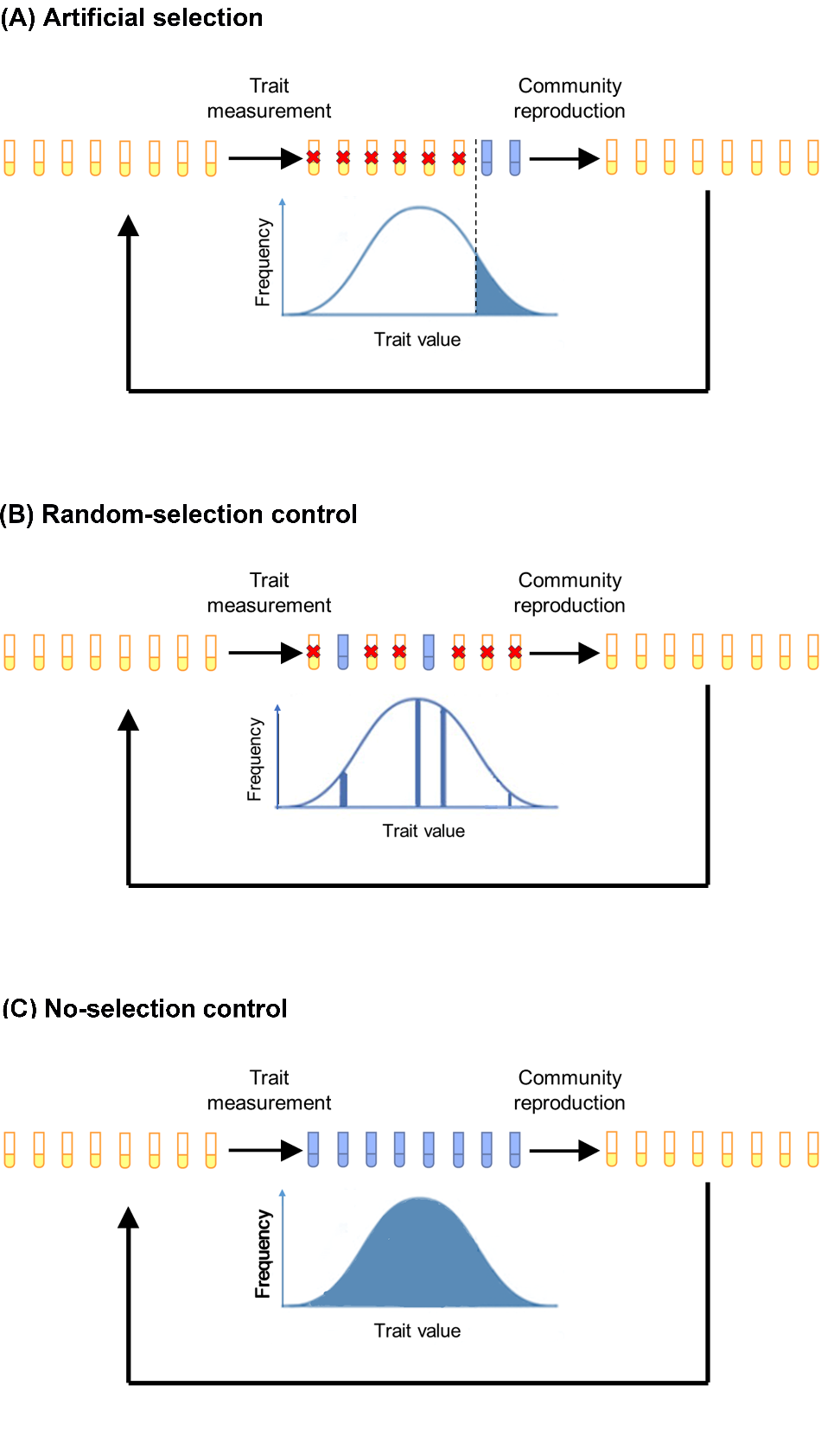


**Figure S1.** A graphical illustration of the artificial selection (A), random-selection control (B) and no-selection control (C). The random-selection protocol chooses a proportion of communities randomly (regardless of their traits) to contribute to the next generation of communities. Under the no-selection control, every community would contribute to one offspring community at each round of community propagation.

**
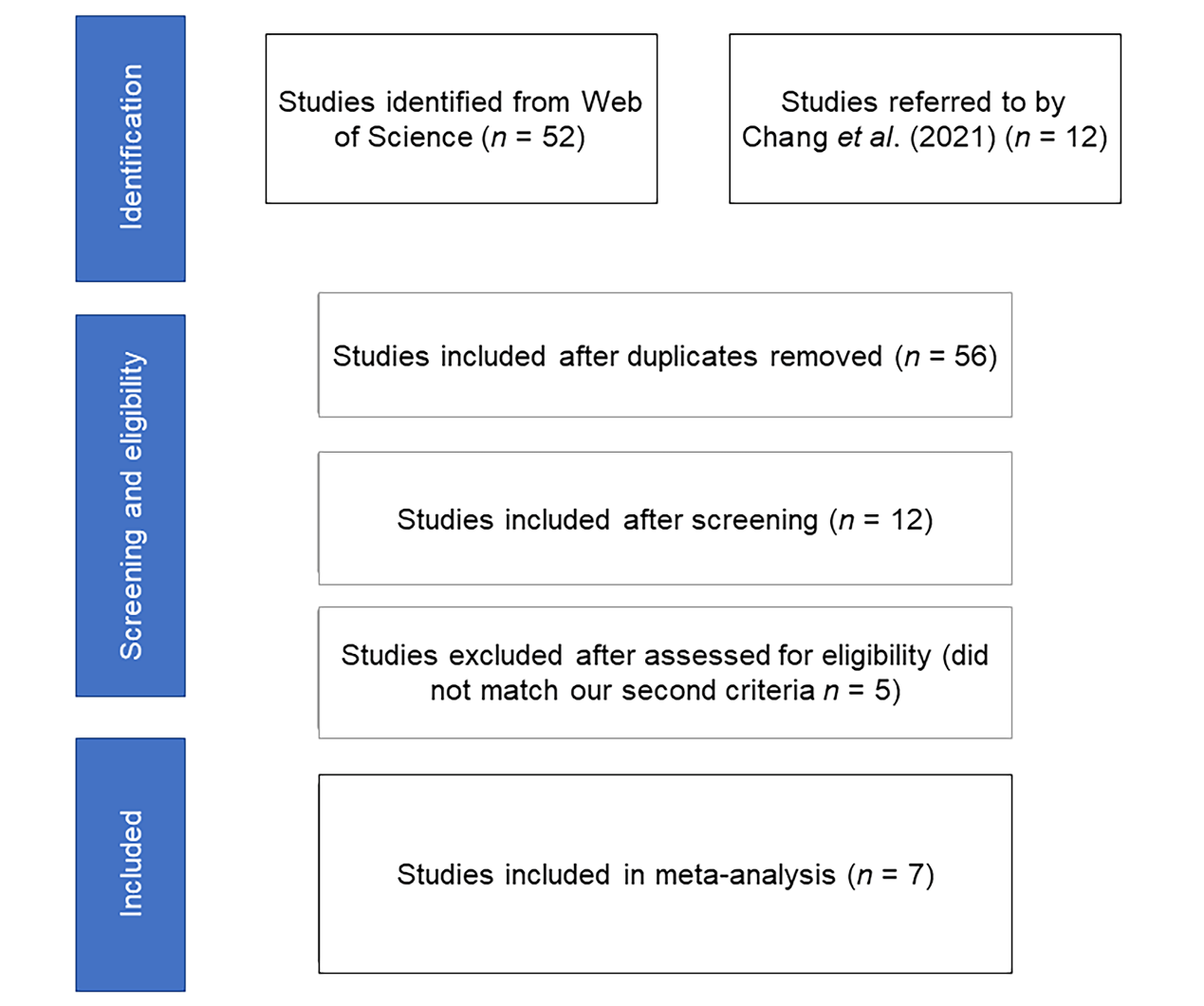
**

**Figure S2.** Preferred Reporting Items for Systematic reviews and Meta-analysis (PRISMA) diagram that shows an overview of the study selection process for our meta-analysis.


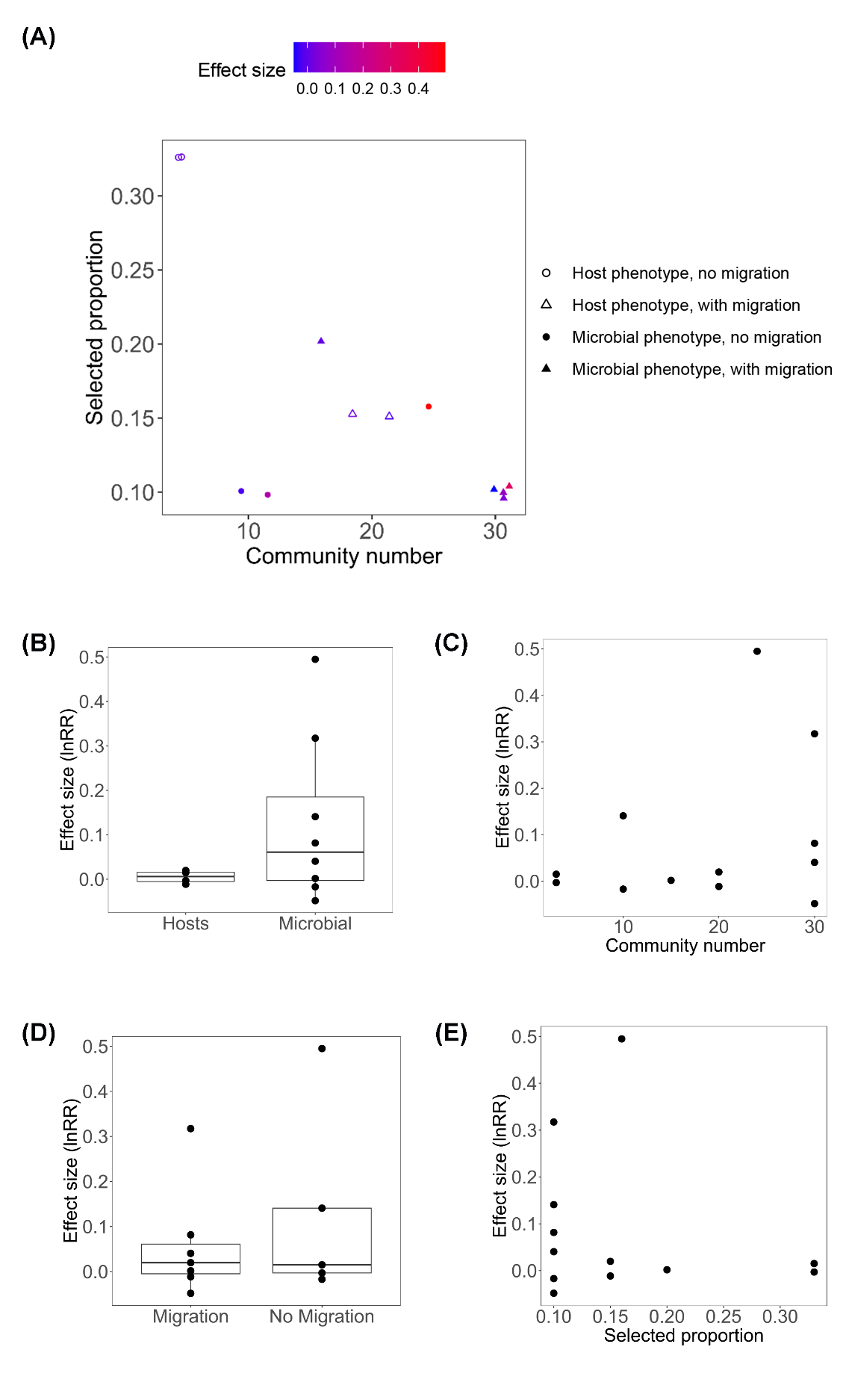


**Figure S3.** Relationship between effect sizes of experiments and the four moderators after an outstandingly large effect size (Chang et al. (2020): cross-feeder) was excluded.
